# Depression, Sensation Seeking, and Maternal Smoking as Predictors of Adolescent Cigarette Smoking

**DOI:** 10.1100/tsw.2006.128

**Published:** 2006-06-12

**Authors:** Judy van de Venne, Kay Bradford, Catherine Martin, Megan Cox, Hatim A. Omar

**Affiliations:** ^1^ Department of Family Studies, University of Kentucky, Lexington, KY 40536-0284, USA; ^2^ Department of Psychiatry University of Kentucky, University of Kentucky, Lexington, KY 40536-0284, USA; ^3^ Department of Pediatrics, J422 Kentucky Clinic, University of Kentucky, Lexington, KY 40536-0284, USA

**Keywords:** smoking, adolescents, depression, sensation seeking, United States

## Abstract

The purpose of this study was to examine maternal and adolescent depression, maternal and teen sensation seeking, and maternal smoking, and their associations with adolescent smoking. Data were collected from a sample of 47 male and 66 female adolescents (ages 11—18 years) and their mothers from three different health clinics. The findings indicated that maternal sensation seeking was linked indirectly with adolescent smoking through teen sensation seeking, both of which were significantly associated with teen smoking (β = 0.29, *p* < 0.001 and β = 0.32, *p* < 0.001, respectively). Teen depression was associated positively with teen smoking (β = 0.24, *p* < 0.01) when controlling for sensation seeking behaviors. Maternal smoking was also directly linked to adolescent smoking (β = 0.20, *p* < 0.05). These findings underscore a potentially important role of sensation seeking in the origins of adolescent smoking, and clarify pathways of influence with regard to maternal attitudes and behaviors in subsequent teenage nicotine use.

## INTRODUCTION

Tobacco use has long been identified as the cause of the greatest amount of preventable disease, disability, and death in the U.S.[1]. Described as a pediatric epidemic, tobacco is considered a gateway drug to other drug use and a robust risk factor in nontobacco substance abuse disorders in later adolescence when nicotine or substance abuse is initiated before 13 years of age[2,3,4]. Each day, more than 6,000 teens under 18 years of age try their first cigarette, and more than 3,000 become daily smokers[5].

The risks inherent to smoking make it imperative to identify the variables associated with the initiation and continuation of smoking, and to clarify the salience of each. The smoking behavior of parents and siblings alike has been linked to both experimental and habitual smoking[6,7]. Adolescent depression is also an important correlate of smoking, particularly for girls[8,9]. Sensation seeking — the pursuit of varied, novel, or intense sensations and experiences — has been studied relatively less often, although there is literature that substantiates linkages between risk-taking behavior and smoking acquisition[10,11,12]. Among adolescents' developmental contexts, the family tends to be the most influential and, therefore, the context of family process is important to address with regard to adolescent smoking[13]. In this study, we examine maternal and adolescent depression, maternal and teen sensation seeking, and maternal smoking to assess the relative strength of their associations with adolescent smoking.

## Depression, Sensation Seeking, and Maternal and Teen Smoking

The association between depression and smoking is well established[9,14]. Because these linkages are correlational, there are efforts underway to understand whether depression leads to smoking or vice versa[8]. In the context of adolescent smoking, the etiology of teen depression is important to clarify. It is known that exposure to maternal depression coupled with marital conflict puts teens at risk for internal and external depressive symptoms and several forms of misconduct, including greater tobacco smoking frequencies in both boys and girls[15,16]. Hammen, Burge, and Adrian[17] found that, in a sample of depressed children and adolescents ages 8–16 years of age, the onset of their symptoms predictably followed their mother's depressive episodes. Children under high stress with nonsymptomatic mothers were less depressed than those with symptomatic mothers[17].

The self-medication of depression may be a reason why people of different ages, including adolescents, smoke[18]. Nicotine is known to enhance cognitive functioning and it can relieve some of the stress inherent to depression by releasing dopamine in the forebrain[19,20]. The antidepressant effects of nicotine may precipitate the use of other drugs to replace its antidepressive effects in cases where smoking is discontinued[21].

Sensation seeking is a predictor of both positive and negative risk behavior among adolescents[22]. Sensation seeking is defined by Zuckerman as the seeking of varied, novel, complex, and intense experiences, coupled with a willingness to take physical, social, legal, and financial risks to have such experiences[10,23]. This trait is associated with relatively earlier age of onset of drug use and abuse, and is a predictor of drug use in adolescence and adulthood[24,25,26]. One study of early and middle adolescents reported higher incidences of sensation seeking for nicotine and alcohol users, and in males who reported marijuana use[27]. Sensation seeking tends to peak in adolescence, particularly in boys, then declines thereafter[10]. In one study, late pubertal onset was associated with decreased alcohol use and lower sensation seeking in males, and the interaction between early pubertal onset and high sensation seeking predicted cigarette use in females[28]. Martin and colleagues also found a positive association with pubertal development and drug use in males and females, controlling for age[27]. Sensation seeking may be more prevalent among whites than among African Americans[10].

There is very little literature addressing the antecedents of sensation seeking, particularly with regard to familial influences. However, Zuckerman posited that parents relatively higher in sensation seeking are likely to encourage sensation seeking exploration in their children[10]. This position is consonant with the tenants of social learning theory, which suggest that observational learning is a key predictor of behavior; thus, ideas derived from observed behavior are likely to lead to enactment of the observed behavior[29]. Theoretically, an adolescent might observe a parent's sensation seeking behaviors (with the reward anticipation, the behavior itself, and any consequences) and may try the same behaviors and attitudes.

Modeling as an antecedent of smoking behavior has received considerable empirical support. It is clear that teens who report more friends who smoke are more likely to smoke[30]. Family influences are also important. Building on evidence that permissive parental drug attitudes are positively associated with drug use, it is known that the smoking behavior of parents, and siblings as well, is associated with smoking initiation and habituation[6,31,32]. In a study of adolescent depression and social introversion, parental smoking status, and residential status, the smoking status of parents explained more variance in adolescent smoking than did the other variables, controlling for the significant effect of age[33].

Likewise, strong maternal antismoking attitudes were shown in another study to be associated with an approximately 50% reduction in rates of adolescent smoking[34].

Teen depression, teen sensation seeking and risk behaviors, and exposure to parental smoking behavior have all been linked separately to teen smoking in past studies. The extent to which these variables remain significantly predictive of smoking when examined together is not known. For example, although maternal depression is associated with depression in adolescents, the potential impact of maternal depression on teen smoking is less clear[35,36]. Moreover, relatively little is known regarding the relationships between sensation seeking and its part in family processes. The goal of this study was to simultaneously examine the associations between mother's and teen's depression, sensation seeking, and adolescent lifetime smoking status. Using structural equation modeling (SEM) to allow simultaneous estimation of these variables[37], we analyzed depression, sensation seeking, and smoking status among mother and adolescent dyads, to examine their associations with adolescent smoking.

Based on past research findings, we hypothesized positive relationships between maternal depression scores and teenage depression scores, between maternal sensation-seeking scores and teenage sensation-seeking scores, and between maternal lifetime smoking status and teenage lifetime smoking status. We also expected to find a positive relationship between adolescent depression levels and lifetime smoking status, and finally, a positive relationship between teenage sensation-seeking scores and teenage lifetime smoking status.

## METHODS

A convenience sample was taken from three area-wide health centers in Lexington, Kentucky: the Family Care Center (FCC), the Kentucky Clinic Department of Pediatrics (PEDS), and the University of Kentucky, Department of Psychiatry (PSYCH). Participants were adolescents, ages 11–18 (mean 14.0, SD 2.02), and one of their parents or a legal guardian. Data from the 113 mother-child dyads were used in this analysis. Of these adolescents, 47 were male and 66 were female. In terms of ethnic origin, 75.2% were white and 24.8% were African American or of other ethnic origin.

### Measures

For this study, the independent variables of maternal depression, maternal sensation seeking, maternal lifetime smoking status, teenage depression, and teenage sensation seeking were employed along with the dependent variable of teenage lifetime smoking status. Control variables such as adolescent age, gender, ethnicity, and referral source were also analyzed.

Maternal depression was measured using the Beck Depression Inventory (BDI)[38]. Sample items include "I do not feel sad" to "I am so sad or unhappy that I can't stand it" and "I don't feel disappointed in myself" to "I hate myself". This instrument has high internal consistency with Cronbach's alphas for the original instrument ranging from 0.76–0.95 and validity correlations from 0.55–0.96 of clinical ratings of depression. For this study, 13 items were used and a mean score calculated where depression was measured as follows: 0 = nonpresent, 1 = mild, 2 = moderate, and 3 = severe. The Cronbach's alpha for these items was 0.89.

Teenage depression (TD) was measured using the Child Depression Inventory Short form (CDI-S) developed for children ages 7–17[39]. The CDI has 27 items in its entirety, but the use of the 10-item 0- to 30-point scale brings comparable results. Sample items include "I am sad once in a while" to "I am sad all the time" and "I do not feel alone" to "I feel alone all the time". Internal consistency of the original instrument ranged from 0.71–0.89 and test-retest coefficients range from 0.74–0.83, respectively. Interpretation of these scores consisted of a mean composite score of items from 0–3. The Cronbach's alpha for these items was 0.83.

Maternal sensation seeking (MSS) was assessed Sensation Seeking Scale (SSS) form V designed and revised by Zuckerman[10]. This form uses a 40-item, forced-choice format. A total score from this scale was used. Internal reliabilities of the Total score on form V ranged from 0.83–0.86. The Cronbach's alpha for this sample was 0.70.

Teenage sensation seeking (TSS) was measured using Russo's revised version of the SSS for children (SSS-C)[40]. This form is considered the best form available for use with children. This version had higher internal reliabilities of 0.83 for the Total score, 0.81 for Thrill and Adventure Seeking, 0.72 for Drug Alcohol Attitudes, and 0.67 for Social Disinhibition. Test-retest reliabilities were moderate in one sample and low in another. The Cronbach's alpha for these items was 0.83.

The dependent variable of Lifetime Smoking Status (LSS) was assessed by utilizing the lifetime use question on the D.A.R.E. Substance Use Questionnaire developed by Clayton, Cattarello, and Johnstone to assess the efficacy of Drug Abuse Resistance Education on teenage drug habits. It specifically states, "How many cigarettes have you smoked in your whole life?"[41].

## RESULTS

The bivariate correlations, means, and standard deviations are reported in Table 1. Teenage lifetime smoking status was significantly associated with teenage depression ($r = 0.24$, $p < 0.05$), teenage sensation seeking ($r = 0.31$, $p < 0.01$), maternal lifetime smoking status ($r = 0.21$, $p < 0.05$), and age ($r = 0.20$, $p < 0.05$); these were all in the expected directions. Teenage depression was significantly higher for girls ($r = 0.19$, $p < 0.05$) and for youth referred through Psychiatry vs. Pediatrics and the Family Care Center ($r = –0.22$, $p < 0.05$). Teenage sensation seeking was significantly associated with maternal sensation seeking in the expected positive direction ($r = 0.34$, $p < 0.01$) and, as expected, was higher in males ($r = –0.31$, $p < 0.01$) and for white respondents ($r = –0.22$, $p < 0.05$).

Structural Equation Modeling (SEM; AMOS v. 4) was then used to test the multivariate pathways. This analysis yielded two separate models. The first model, the Full Effects Model, simultaneously assessed the direct relationships of all key and control variables. Second, the Trimmed Full Effects Model tested these same variables with all nonsignificant structural pathways removed. Standardized coefficients are reported in Table 2 (see also Fig. 1).

In the Trimmed model, maternal sensation seeking was linked positively with teenage sensation seeking ($\beta = 0.29$, $p < 0.001$), and teenage sensation seeking was significantly associated with teenage lifetime smoking ($\beta = 0.32$, $p < 0.001$). Teen sensation seeking was higher in males ($\beta = –0.25$ $p < 0.01$), in white respondents ($\beta = –0.21$, $p < 0.05$), and increased with age ($\beta = 0.20$, $p < 0.05$). Teenage depression was a significant predictor of teen lifetime smoking ($\beta = 0.24$, $p < 0.01$). Higher levels of depression were observed in older teens ($\beta = 0.21$, $p < 0.05$) and those from the psychiatric referral source ($\beta = –0.26$ $p < 0.01$) in this model. Maternal lifetime smoking was also a significant predictor of teenage lifetime smoking ($\beta = 0.20$, $p < 0.05$). The association between referral source and teen smoking was initially significant ($\beta = 0.20$, $p < 0.05$), but became insignificant in this model. The fit for both models was adequate; indices are reported in Table 3.

## DISCUSSION

The purpose of this study was to conduct a test of the multivariate relationships between maternal and teen depression, sensation seeking, and lifetime smoking statuses. The findings were largely supportive of our hypotheses and of previous works in this field regarding teenage nicotine use. The existence of direct linkages between maternal lifetime smoking status, teenage depression, and teenage sensation seeking with positive teenage lifetime smoking status supports the premise that youth may be influenced by the modeling behaviors of their parents, when vulnerable to depression or needs for high sensation, as suggested by social learning theory and as supported by past literature[10,29,42].

**TABLE 1**
**Bivariate Correlations**

| Variables | 1 | 2 | 3 | 4 | 5 | 6 | 7 | 8 | 9 | 10 |
|---|---|---|---|---|---|---|---|---|---|---|
| 1. Teenage lifetime smoking status | 1 | | | | | | | | | |
| 2. Teen depression | 0.24* | 1 | | | | | | | | |
| 3. Teen sensation seeking | 0.31** | –0.03 | 1 | | | | | | | |
| 4. Maternal lifetime smoking status | 0.21* | 0.12 | –0.02 | 1 | | | | | | |
| 5. Maternal depression | 0.14 | 0.15 | –0.09 | 0.17 | 1 | | | | | |
| 6. Maternal sensation seeking | 0.097 | 0.08 | 0.34** | 0.09 | 0.20* | 1 | | | | |
| 7. Age | 0.20* | 0.17 | 0.12 | 0.05 | –0.02 | –0.05 | 1 | | | |
| 8. Gender | 0.01 | 0.19* | –0.31** | 0.11 | 0.05 | –0.26** | 0.20* | 1 | | |
| 9. Race | –0.04 | –0.09 | –0.27* | 0.01 | 0.08 | 0.01 | 0.000 | 0.03 | 1 | |
| 10. Referral | –0.16 | –0.22* | –0.00 | 0.15 | –0.11 | 0.01 | 0.18 | 0.09 | 0.15 | 1 |
| *Mean* | 0.541 | 0.243 | 11.734 | 1.411 | 0.525 | 5.469 | 14.00 | 0.584 | 0.248 | 0.496 |
| *Standard Deviation* | 0.807 | 0.310 | 4.551 | 0.844 | 0.634 | 3.536 | 2.040 | 0.495 | 0.434 | 0.502 |

*$p < 0.05$; **$p < 0.01$; Gender: 0 = male, 1 = female; Race: 0 = white, 1 = black or other; Referral source: 0 = Psychiatry, 1 = Pediatrics and Family Care Center.

**TABLE 2**
**Structural Path Coefficients†**

| | Teen Depression | Teen Sensation Seeking | Teen Lifetime Smoking Status |
|---|---|---|---|
| Teen depression | | | (0.18*) 0.21* |
| Teen sensation seeking | | | (0.33***) 0.32*** |
| Teen lifetime smoking status | (0.18*) 0.21* | (0.33***) 0.32*** | |
| Maternal sensation seeking | | (0.29***) 0.29*** | |
| Maternal lifetime smoking status | | | (0.21**) 0.22** |
| Maternal depression | (0.14) | | |
| Gender | (0.16) 0.17 | (–0.25**) –0.26** | (0.04) |
| Age | (0.18*) 0.21* | (0.19*) 0.19* | (0.15) |
| Ethnicity | (0.06) | (–0.21*) –0.21** | (0.10) |
| Referral source | (–0.25**) –0.26** | (0.02) | (–0.20*) –0.15 |

*$p < 0.05$; **$p < 0.01$; ***$p < 0.001$; ( ) Path coefficients prior to trimming; †All coefficients are standardized; Gender: 0 = male, 1 = female; Race: 0 = white, 1 = black or other; Referral source: 0 = Psychiatry, 1 = Pediatrics and Family Care Center.

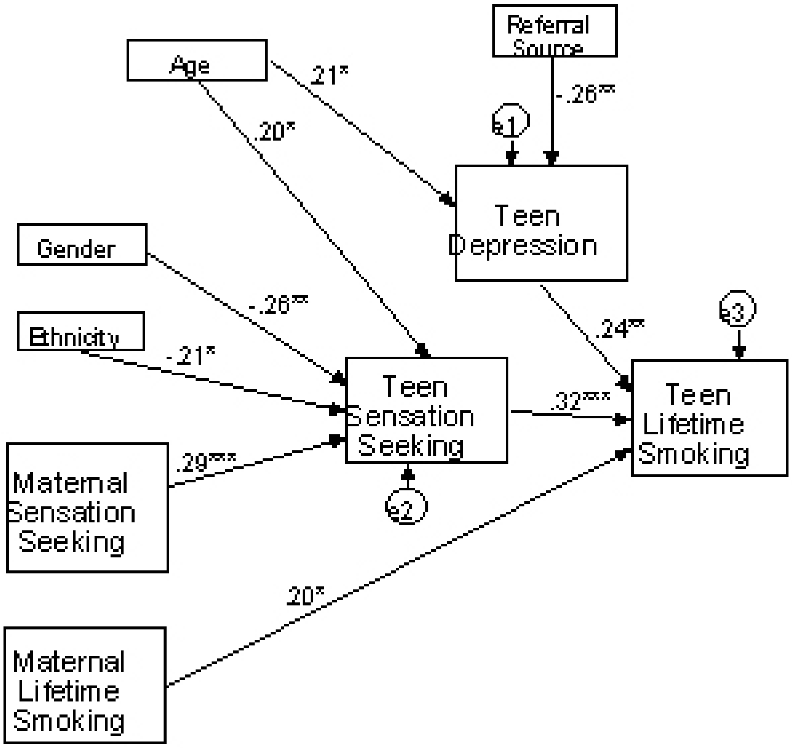

**FIGURE 1.** Trimmed Full Effects Model (standardized coefficients). Coefficients indicate when the predictor variable increases 1 standard deviation, the dependent variable increases (e.g., by 0.29 standard deviations); $*p < 0.05$, $**p < 0.01$, $***p < 0.001$; all coefficients are standardized: Gender, 0 = male, 1 = female; Race, 0 = white, 1 = black or other; Referral source: 0 = Psychiatry, 1 = Pediatrics and Family Care Center.

**TABLE 3**
**Model Fit Indices**

| Fit Measures | Full Effects Model | Trimmed Full Effects Model |
|---|---|---|
| $R^2$ | 22.3 | 22.4 |
| $X^2$ | 23.311 | 40.402 |
| *Df* | 23 | 27 |
| *p* | 0.443 | 0.047 |
| Normed Fit Index | 0.984 | 0.970 |
| Relative Fit Index | 0.961 | 0.950 |
| RMSEA | 0.011 | 0.047 |

The indirect link between maternal sensation seeking and teenage lifetime smoking via teenage sensation seeking is an important finding. Maternal sensation seeking was not *directly* related in any analysis to teenage smoking, but the significant indirect link underscores the importance of maternal influence in adolescent smoking behavior. This is of particularly import because the strength of these associations remained, even when both maternal and teen depression were included in the model.

Zuckerman suggested that a general sensation-seeking trait might be biologically based, but in addition, that high sensation-seeking parents might also encourage expression of high-risk behavior in their children[10]. The observed impact of maternal sensation seeking on teen sensation seeking, and subsequently on teen smoking, confirms this notion and provides clarification of the influence of maternal sensation seeking relative to maternal smoking.

The significant relationships in this study between sensation seeking and gender, age, and ethnicity are supported by results from past literature. Similar to our finding that Caucasians and males were higher in sensation seeking, Zuckerman's review suggests that white males tended to be higher in sensation seeking than females and minorities, and that males tended to peak in sensation seeking in adolescence[10]. Together, these results suggest a greater vulnerability in white adolescent males toward sensation seeking, and thus potentially toward smoking.

Depression was found to predict smoking, and was observed to increase with the age of the adolescent. This is consistent with Angold and Rutter, who found increasing levels of depression with age in adolescent boys and girls, with full expression in girls around 16 years of age[42]. The lack of relationship between depression and gender might possibly have been due to the relatively lower mean age of 14 years of adolescents in our study. Higher levels of depression in the psychiatric group were possibly due to innate differences in those seeking psychiatric services vs. pediatrics and family care. They also had a higher level of lifetime smoking, suggesting that nicotine use may be a method to self-medicate depressive feelings[18].

One surprising finding was that maternal depression was not significantly related with any other variable, even in the bivariate analysis. The coefficients of 0.14 (maternal depression and teen smoking) and 0.15 (maternal depression and teen depression) suggest trends in these variables being related. Maternal depression has clear links in prior literature to both teen smoking and particularly to teen depression[15,44]. The lack of relationship in this study may possibly be due to low sample size.

There are several limitations to this study. First, a convenience sample might create bias in results[45]. This sample was gathered from three clinical settings and so is not representative of the overall adolescent population. Also, Kentucky's youth are higher than the national average in teenage smoking in middle school and high school populations[5], which may also limit the generalizability of this sample. In addition, the small sample size may affect the reliability of a study by yielding coefficients that are either too high or too low[46]. And finally, the correlational design does not explain causal effects between depression, sensation seeking, and smoking.

Despite limitations, these findings may enable a more comprehensive discussion of pathways to smoking status throughout adolescence and may help to guide interventions for health and social service professionals. Preventative interventions may include the use of adult and child sensation-seeking scales to assess for risk attitudes, and child depression inventories to assess for possible vulnerability to parent- and peer-modeled smoking behaviors. Teens with high sensation-seeking scores may be encouraged to take up alternative behaviors like school sports or adventurous hobbies like hiking or skiing that satisfy these needs without the negative consequences of smoking[22]. Nonsmoking teens with high depression scores may be advised to undergo counseling or other forms of treatment to offset their vulnerability to the smoking influences of others or their need to medicate their depression with nicotine. These findings also suggest the value of assessing the relative risks from maternal sensation seeking and smoking in the prevention of teenage smoking.

For Kentucky adolescents and their parents, future research is needed in several areas. One task is to develop valid and reliable instruments to test cultural influences on adolescent risk attitudes towards smoking in states like Kentucky. In Kentucky, higher proportions of youth are current or established smokers than U.S. youth[5] and in 2002, Kentucky adult populations age 18 years and over had the highest prevalence of current smoking in the nation, according to Behavioral Risk Factor Surveys (BRFS)[46]. What messages are given by media role models, family, and community members at large concerning tobacco use as risky behavior? Which messages have stronger influences on an adolescent's perception of smoking as a risk behavior? Researchers note that parental communication of clear nonsmoking messages and consistency in rule setting are two important avenues to tobacco prevention[5].

Could a lack of these clear messages and boundaries, along with permissive attitudes toward smoking by the family or culture at large, increase a Kentucky teenager's likelihood of cigarette experimentation?

Finally, though the results indicated no relationship between maternal depression and teen depression or teen lifetime smoking status, it may be that this is a peculiarity of this sample in terms of composition or relatively small size, given that these linkages are often found in other studies. Larger numbers of subjects and the use of random samples may yield very different results than those found in this study. Parental depression may lead to a lack of parental support or involvement, lack of consistent rule setting, and may be a factor behind parental lifetime smoking status[17,18]. For prevention programs to be effective, we need definitive answers in these areas and clear strategies to combat negative moods and/or risky cognitions that influence an adolescent's intentions to smoke.

## ACKNOWLEDGMENTS

This study was supported by National Institute on Drug Abuse grants 1 KO8 DA00333 and P50 DA05312 to Catherine Martin.

## BIOSKETCHES

**Judy van de Venne, MS,** is currently a doctoral student at the University of Kentucky. She holds a Master of Science in family studies. Her interests include maternal depression and child outcomes and intergenerational transmission of domestic violence and substance abuse. E-mail: jgvalo2@uky.edu

**Kay Bradford, PhD, LMFT,** is an assistant professor and director of Marriage and Family Therapy at the University of Kentucky. He holds a Master of Science in family studies/marriage and family therapy from the University of Maryland and a doctorate in marriage, family and human development from Brigham Young University. His interests include couple conflict and adolescent well-being, and psychotherapist training. E-mail: kbrad@uky.edu

**Catherine A. Martin, MD**, Professor and Vice Chair for Research, Department of Psychiatry, Child Psychiatry Division, University of Kentucky, Lexington. Her clinical and research interests are in the area of Attention Deficit/Hyperactivity Disorder and nicotine dependence in adolescents and adults. E-mail: cmartin@uky.edu

**Megan Cox, MA**, is currently the project coordinator for the third-party evaluation of the KIDS NOW Initiative in Kentucky. She holds a Master of Arts degree from Marshall University in sociology and is completing her PhD in family studies at the University of Kentucky. Her interests include inequality among early childhood professionals and identity development among biracial youth. E-mail: mecox2@uky.edu

**Hatim A. Omar, MD**, Professor of Pediatrics, Obstetrics and Gynecology and Director of the Section of Adolescent Medicine, Department of Pediatrics, University of Kentucky, Lexington. He is the recipient of the Commonwealth of Kentucky Governor's Award for Community Service and Volunteerism. He is an expert in adolescent medicine and pediatric and adolescent gynecology. E-mail: haomar2@uky.edu
